# Supplementary material for: A systematic approach to orient the human protein–protein interaction network
Source: Nat Commun. 2019 Jul 9;10:3015. doi: 10.1038/s41467-019-10887-6 (PMC6617457; doi:10.1038/s41467-019-10887-6)
Supplement: Supplementary file 1 — Supplementary Information [file 41467_2019_10887_MOESM1_ESM.pdf]

A systematic approach to orient the human protein-protein interaction network

Supplementary Information

Silverbush et al.

Supplementary Note 1. Formal description of the logistic regression classifier

The classifier receives as input labeled data *(x,y)*, where *x* denotes a feature vector (with a 1-entry as its first feature) for any given interaction and *y* denotes whether its orientation is True (1) or False (0). The classifier models the posterior probability of the label as a logistic function of the features:

$$P(y|x) = logit(w \cdot x) \tag{1}$$

and aims to learn a weight vector *w* that maximizes the product of these probabilities, across all training data points. For regularization, we substract a regularization term from this optimization criterion $\gamma \sum_i |w_i|$, where γ is set via a nested cross validation.

Supplementary Note 2. Use of known orientations by D2D

Our D2D method has two conceptual steps: (i) scoring edge directions using diffusion, and (ii) training a classifier that is fed with these scores. Prior knowledge on the orientation of a subset of the edges can be used in each of the steps, either to guide the diffusion or to train the classifier. However, the same edge cannot be used by both steps, as directing it while diffusing would mean that we cannot use its scores in the opposite direction as features for the classifier training. Thus, we used direction information only in the second step of classifier training.

In order to support this choice, we tested the use of known directions in the diffusion process as well. To this end, we focused on the STKE test set which has the lowest AUPR compared to the other four test sets (KPIs, PDIs, EGFR and E3). First, we used the known orientations of the other four datasets during the diffusion and left STKE unoriented for training the classifier. This approach yielded the same results as our original approach, possibly due to the sparsity of directed edges in the network. Second, we partitioned the STKE edges to 2/3 that remained directed during the diffusion, and 1/3 that were used for training (in a 3-fold cross validation). This approach yielded slightly worse results (AUPR 0.71 when oriented with drug

response data). As these versions require extra diffusion computations (since different parts of the network are kept directed every time) and did not improve the results, we opted to use the direction information only in the training phase.

Supplementary Note 3. Construction of drug-specific differentially expressed genes signatures

For completeness, we briefly describe the normalization and filtering procedures of Iskar et al. [1] for drug-specific signature differentially expressed genes construction. In order to compute a set of differentially expressed genes per drug, we employed two steps. In the first step we computed a set of differentially expressed genes per drug and cell line, by z-scoring all genes' expression in the drug microarray and retaining only the statistically significant genes (FDR 0.01). In the second step, we created a set of differentially expressed genes per drug by taking the union of the three cell line sets.

Supplementary tables

Supplementary Table 1. AUPR results for the 5 unbiased test sets.

		AUPR results						
**Instances (50% correct orientations + 50% incorrect orientations)**	**Source**	**Drug response**	**Breast**	**Colon**	**AML**	**Ovarian**	**Benchmark orientation (topology only)**	**Vinayagam**
KPIs: 3,596 kinase-substrate and phosphatase-substrate interactions, filtered to avoid degree bias	www.phosphosite.org[2]	0.92	0.89	0.85	0.90	0.89	0.62	0.64
PDIs: 342 protein-DNA interactions, filtered to avoid degree bias	ChEA database: integrating genome-wide ChIP-X experiments[3]	0.90	0.90	0.84	0.92	0.88	0.71	0.72

STKE: 900 signal-transduction interactions in mammalians	Database of Cell Signaling[3] (http://stke.sciencemag.org/cm/	0.74	0.68	0.69	0.71	0.67	0.47	0.71
EGFR: 234 interaction from EGFR pathway	Samaga et al.[4]	0.85	0.73	0.88	0.72	0.81	0.51	0.58
E3: 208 E3 ubiquitination interactions	Du et al.[5]	0.85	0.93	0.94	0.89	0.90	0.49	0.67

A correct orientation reflects the direction of an interaction as annotated by the respected database, while an incorrect orientation is created by taking the opposite direction of the same interaction.

Supplementary Table 2. Summary of large scale D2D orientations.

<table>
<tr><th rowspan="2">Guiding source</th><th colspan="2">Description</th><th rowspan="2">Number of guiding sets</th><th rowspan="2">Number of positive and negative known directed interactions (train)</th><th rowspan="2">Number of directed interactions in the final inferred network</th><th rowspan="2">Number of undirected interactions in the final inferred network</th></tr>
<tr><th>Causes</th><th>Effects</th></tr>
<tr><td>Drug response data</td><td>Drug targets</td><td>Differentially expressed genes in response to treatment with the drug</td><td>644 drugs</td><td rowspan="5">33,756 directed interactions</td><td>148,067</td><td>101</td></tr>
<tr><td>Colon cancer</td><td rowspan="4">Genomic alterations in the tumor</td><td rowspan="4">Differentially expressed genes in the tumor with respect to a matched healthy tissue</td><td>596 patients</td><td>34,209</td><td>113,959</td></tr>
<tr><td>AML</td><td>200 patients</td><td>98,567</td><td>49,601</td></tr>
<tr><td>Breast cancer</td><td>960 patients</td><td>108,702</td><td>39,466</td></tr>
<tr><td>Ovarian cancer</td><td>316 patients</td><td>51,383</td><td>96,785</td></tr>
</table>

Supplementary figures

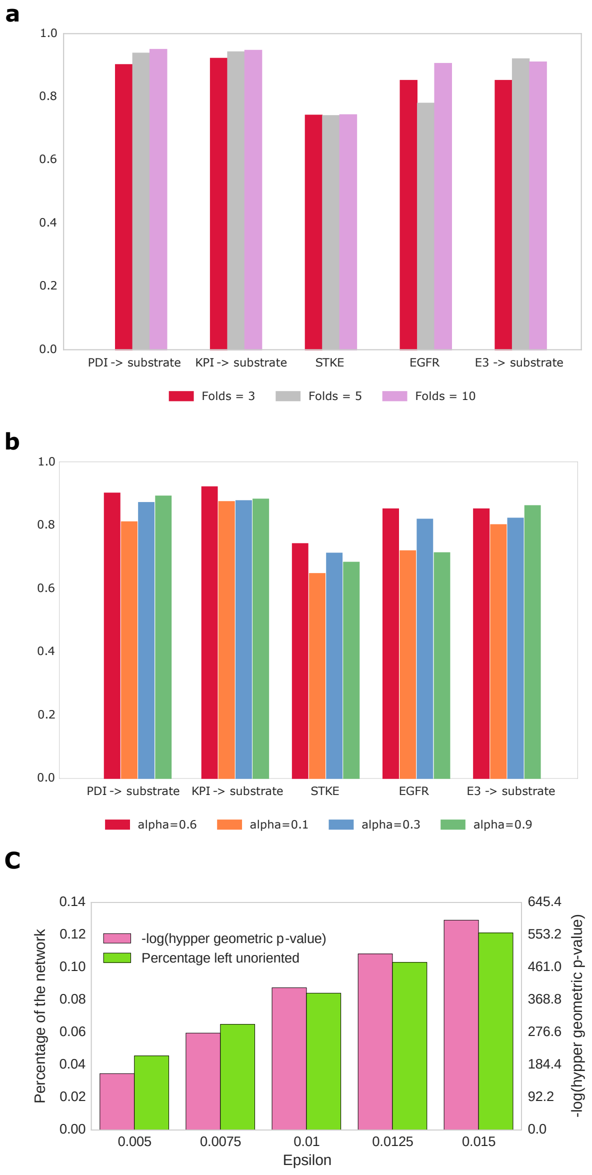

Supplementary Figure 1. Testing parameters robustness shows that the high performance of the method is consistent across a wide range of parameters values. Plotted are AUPR results for the 5 unbiased test sets, orienting the network guided by the drug response data using **(a)** different folds for cross validation (3 is the default value), and **(b)** different alpha values for the diffusion (0.6 is the default value). **(c)** Unoriented interactions are enriched with known complexes across a range of choices for epsilon.

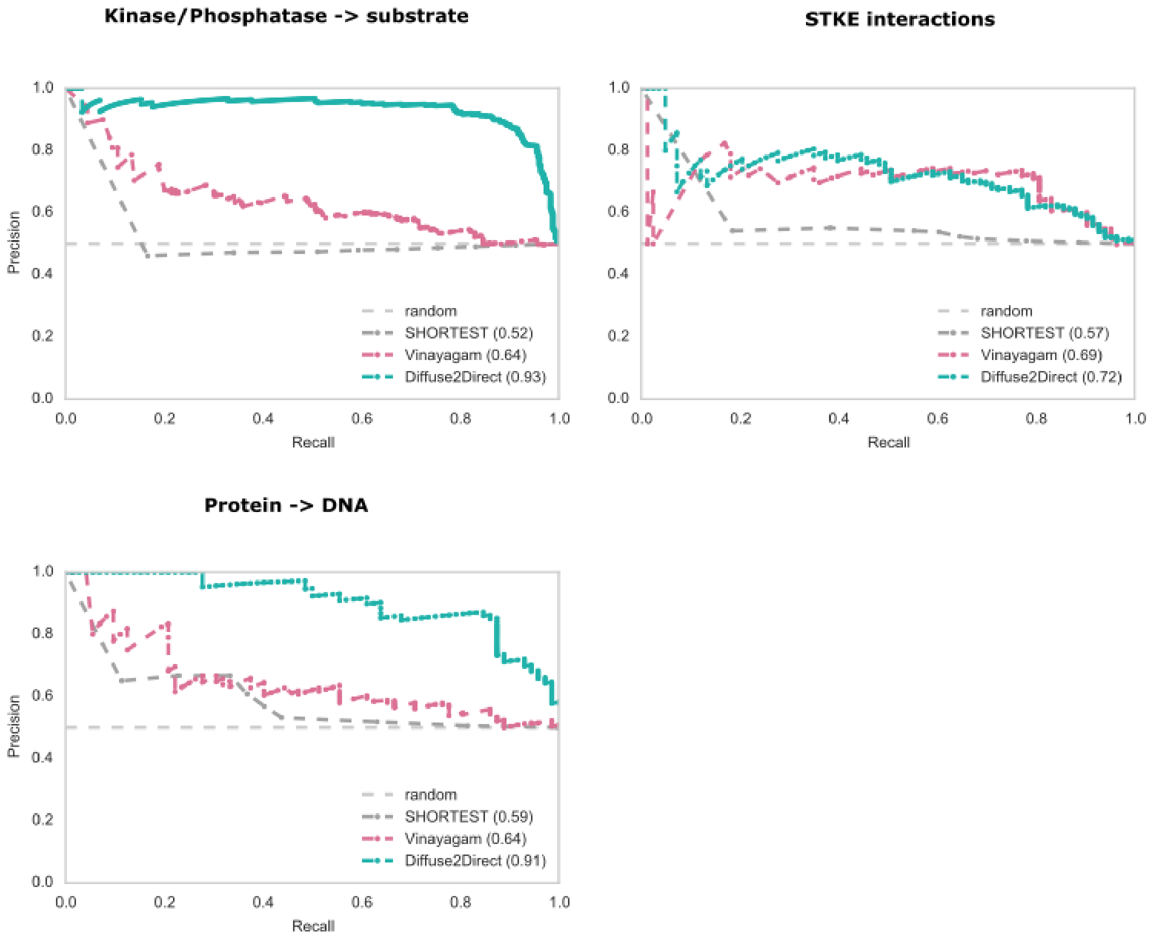

Supplementary Figure 2. Comparison of orientation methods restricted to the interactions covered by SHORTEST**.** Cross validation results comparing the inferred directions to subsets of interactions with known directionality: kinases and phosphatases to their substrates; protein-DNA interactions; and the STKE signaling interactions used for validation by Vinayagam et al.[7]. SHORTEST is limited in coverage and hence was not able to infer directions for smaller subsets such as EGFR pathway and E3 ubiquitination interactions.

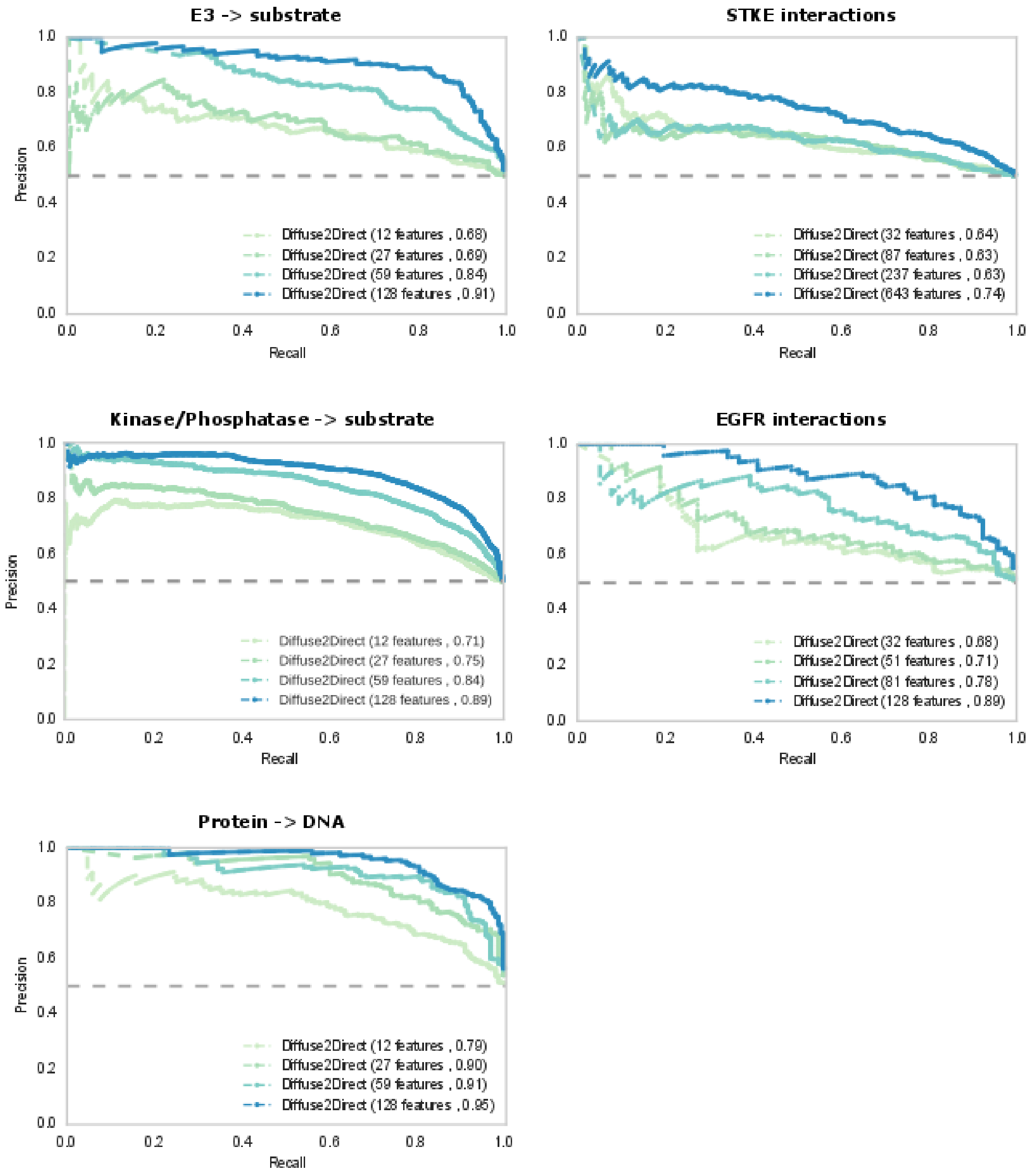

Supplementary Figure 3. Performance as a function of the percentage of drugs guiding the orientation. In this setting only, we used a logistic regression classifier with no regularization in order to manually control the percentage of guiding drugs used.

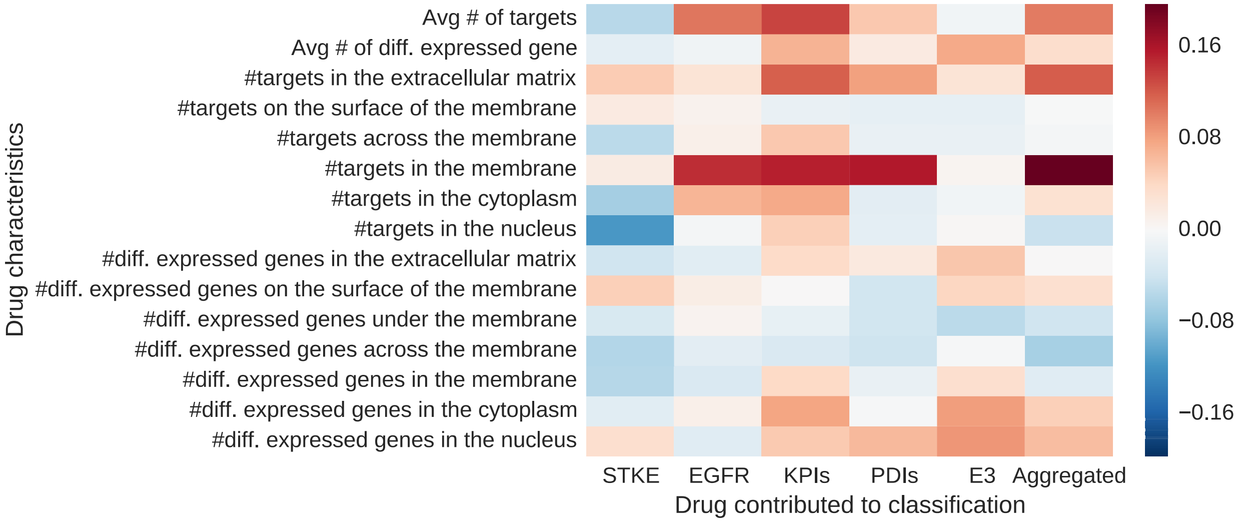

Supplementary Figure 4. Likelihood of a drug to contribute to the orientation.

Demonstrated by Pearson correlation of the number of times a drug was chosen as a feature by the classifier and its attributes, namely 1) number of known drug targets, 2) number of genes that were observed to be differentially expressed in response to treatment with the drug, 3) cellular localization of known drug targets, and 4) cellular localization of the genes that were observed to be differentially expressed in response to the drug.

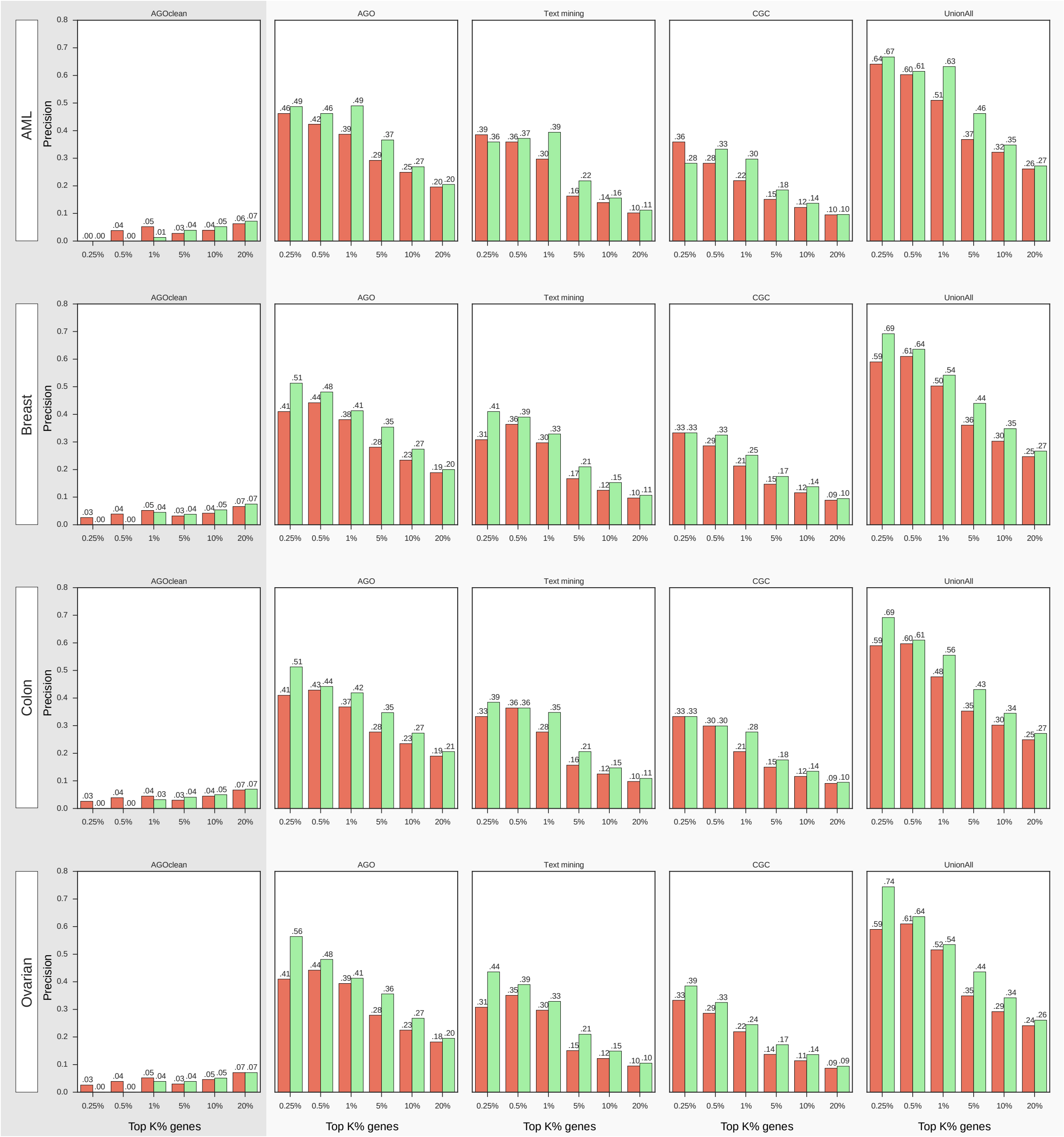

Supplementary Figure 5 (extending Figure 5b). Orientation improves the prediction of drug targets and cancer driver genes. The genes in the network are ranked by their proximity to the differentially expressed genes of the cancer patients. We calculated the percentage of genes out of the top K% ranked genes matching known driver genes and known non-drivers

from multiple sources, ranging from top 0.25% to 20%. The oriented network consistently reports more known driver genes and less known non-driver genes than the unoriented one.

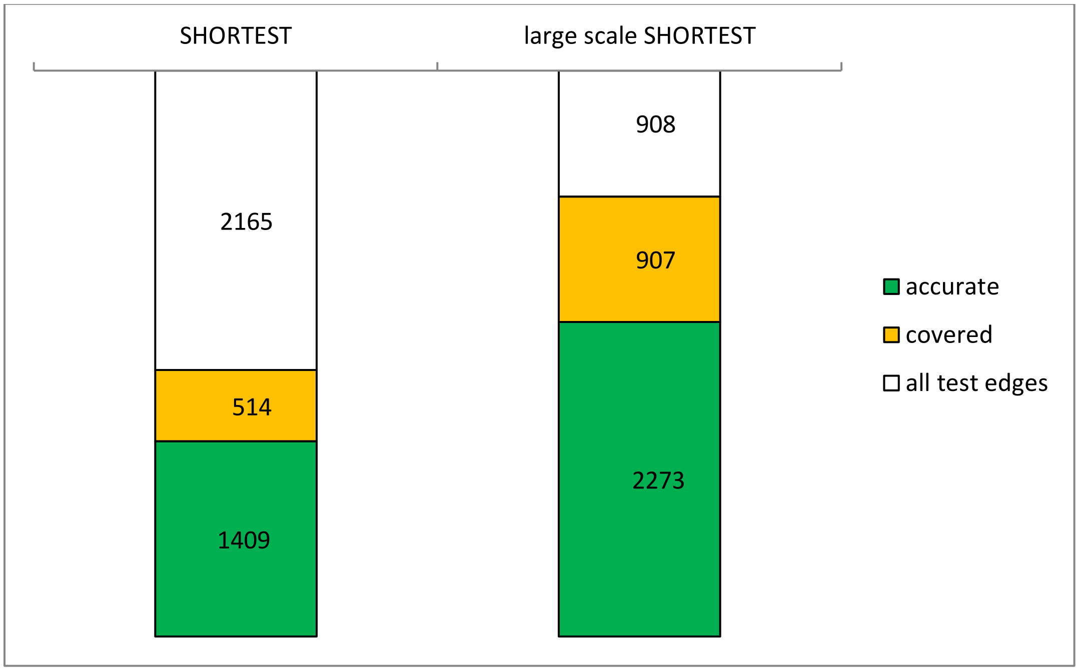

Supplementary Figure 6. Performance evaluation of SHORTEST against its extension. We adapted the SHORTEST method, originally developed for yeast, to make it scalable to the large networks. In yeast, the performance of the adapted method is comparable to that of the original method, with similar accuracy (71% vs. 73%) and higher coverage.

Supplementary References

1. Iskar, M. *et al.* Drug-Induced Regulation of Target Expression. *PLoS Comput Biol* **6**, e1000925 (2010).
2. Hornbeck, P. V., Chabra, I., Kornhauser, J. M., Skrzypek, E. & Zhang, B. PhosphoSite: A bioinformatics resource dedicated to physiological protein phosphorylation. *PROTEOMICS* **4**, 1551–1561 (2004).
3. Lachmann, A. *et al.* ChEA: transcription factor regulation inferred from integrating genome-wide ChIP-X experiments. *Bioinformatics* **26**, 2438–2444 (2010).
4. Database of Cell Signaling, as seen April 23, 2009. Available at: http://stke.sciencemag.org. (Accessed: 16th December 2017)
5. Samaga, R., Saez-Rodriguez, J., Alexopoulos, L. G., Sorger, P. K. & Klamt, S. The logic of EGFR/ErbB signaling: theoretical properties and analysis of high-throughput data. *PLoS Comput. Biol.* **5**, e1000438 (2009).
6. Du, Y., Xu, N., Lu, M. & Li, T. hUbiquitome: a database of experimentally verified ubiquitination cascades in humans. *Database J. Biol. Databases Curation* **2011**, bar055 (2011).
7. Vinayagam, A. *et al.* A directed protein interaction network for investigating intracellular signal transduction. *Sci. Signal.* **4**, rs8 (2011).
