# Supplementary material for: A systematic approach to orient the human protein–protein interaction network
Source: Nat Commun. 2019 Jul 9;10:3015. doi: 10.1038/s41467-019-10887-6 (PMC6617457; doi:10.1038/s41467-019-10887-6)
Supplement: Supplementary file 3 — Description of Additional Supplementary Files [file 41467_2019_10887_MOESM3_ESM.pdf]

Description of Additional Supplementary Files

File Name: Supplementary Data 1
Description: Consensus network.
A consensus oriented network based on the agreement of five source-specific orientations (drug response data, AML, ovarian, breast and colon cancer data). The file contains the following information for each interaction in the network: 1) its inferred direction, such that each edge is directed from source entrez id to target entrez id. Interactions that are inferred to remain undirected will still appear once, but will be inferred as undirected using the fifth field; 2) interaction type, indicating whether the interaction was taken from a previously directed set (sets defined in Supplementary Table 1) or the undirected database BioGRID. If the edge is part of a set that required down-sampling, it will be marked as "balanced" if chosen by the down-sampling process; 3) interaction confidence assigned by the software ANAT reflecting the confidence of the existence of the interaction; 4) orientation confidence assigned by this study and indicating the confidence of the direction inferred for the interaction; 5) An indicator to whether the interaction is inferred to be directed, set according to orientation confidence threshold.
